# Clinical Characteristics and Gene Expression of JAK2, STAT3, miRNA-155, and miRNA-216a in Young Adults with Acute Ischemic Stroke

**DOI:** 10.3390/ijms27041991

**Published:** 2026-02-19

**Authors:** David Vidal-González, Jazmin Marquez-Pedroza, Betsabé Contreras-Haro, Ana Miriam Saldaña-Cruz, Carlos Fernando Godínez-González, Antonio Kobayashi-Gutiérrez, Nayeli Alejandra Sánchez-Rosales, Edgar Ricardo Valdivia-Tangarife, José de Jesús García-Rivera, Idarmis Brisseida Reyes-Cortés, Blanca Miriam Torres-Mendoza, Martha Rocio Hernández-Preciado

**Affiliations:** 1 High Specialty Medical Unit, Department of Neurology, Western National Medical Center of the Mexican Institute of Social Security, Guadalajara 44340, Mexico; davidvidal.001@gmail.com (D.V.-G.); carlos.godinez3662@academicos.udg.mx (C.F.G.-G.); drkoba@hotmail.com (A.K.-G.); naye_ale@hotmail.com (N.A.S.-R.); garciar10@hotmail.com (J.d.J.G.-R.); idarmisreyco@gmail.com (I.B.R.-C.); 2 Neurosciences Division, Western Biomedical Research Center, Mexican Institute of Social Security, Guadalajara 44340, Mexico; jaz180688@gmail.com; 3 Department of Biomedical Sciences, Tonala University Center, University of Guadalajara, Tonala 45425, Mexico; betsabecoha@gmail.com; 4 Biomedical Research Unit 02, Mexican Social Security Institute, Guadalajara 44340, Mexico; 5 Physiology Department, Institute of Experimental and Clinical Therapeutics, University Center for Health Sciences, University of Guadalajara, Guadalajara 44340, Mexico; ana.saldanac@academicos.udg.mx; 6 Department of Neurosciences, University Center for Health Sciences, University of Guadalajara, Guadalajara 44340, Mexico; ricardovaldiviatangarife@outlook.com; 7 Department of Philosophical, Methodological and Instrumental Disciplines, University Health Sciences Center, University of Guadalajara, Guadalajara 44340, Mexico; 8 High Specialty Medical Unit, Pediatric Hospital, Western National Medical Center of the Mexican Institute of Social Security, Guadalajara 44340, Mexico

**Keywords:** ischemic stroke, gene expression, acute ischemic strokes

## Abstract

Acute ischemic stroke (AIS) is a leading cause of long-term disability and death. The genetic, epigenetic, and molecular mechanisms underlying AIS in young adults require further investigation. Inflammatory pathways, such as the programmed death-ligand 1 (PD-L1) and the Janus kinase 2/signal transducer and activator of transcription 3 (JAK2/STAT3) signaling pathway, are implicated in promoting post-ischemic neuroinflammation and neuronal apoptosis. While miR-155 and miR-216a are mediators of inflammation, apoptosis, and tissue repair following AIS. This exploratory study aimed to analyze the expression of the JAK2/STAT3, miR-155, and miR-216a genes in young AIS patients (mean age: 39.4 ± 11.9 years) versus the healthy population (HP). Peripheral blood samples were collected, and gene and miRNA expressions were measured using quantitative real-time PCR. Our results showed that stroke patients exhibited overexpression of all genes (*p* < 0.001), except for miRNA-216a (*p* = 0.061), compared to HP. These findings suggest that JAK2, STAT3, and miR-155 could be potential biomarkers for patients with AIS.

## 1. Introduction

Acute ischemic stroke (AIS) remains a significant global health burden and a leading cause of long-term disability and death across all age groups [1]. However, the most alarming trend is the rising incidence of AIS among young adults, demanding urgent action to prevent early neurological disability and its significant socioeconomic consequences [2].

While many young AIS patients exhibit traditional vascular risk factors such as hypertension, smoking, dyslipidemia, and diabetes, these do not fully explain stroke etiology in individuals under 45 years of age [3,4]. This gap highlights the need to investigate the complex interplay of genetic, epigenetic, and molecular mechanisms underlying AIS in the young [5].

Recent research has highlighted inflammatory pathways, such as JAK2/STAT3 signaling and PDL-1, implicated in promoting post-ischemic neuroinflammation and neuronal apoptosis [6,7]. Meanwhile, dysregulation of non-coding RNAs, particularly microRNAs like miR-155 and miR-216a, has emerged as a key mediator of inflammation, apoptosis, and tissue repair after AIS [8,9].

Understanding these molecular contributors is a critical step toward developing age-specific biomarkers and precision therapies. This ongoing investigation is crucial for enhancing outcomes in young AIS patients, particularly those lacking traditional cardiovascular risk factors [5]. In this context, we hypothesized that AIS in young adults involves the early activation of an inflammatory JAK2/STAT3–miR-155 axis, with increased PD-L1 expression, while miR-216a, being neuroprotective, might be more highly expressed after ischemic injury. Therefore, this study aimed to analyze the expression of JAK2, STAT3, PD-L1, miR-155, and miR-216a in patients with AIS compared to a healthy population (HP).

## 2. Results

Medical records were analyzed from May 2021 to May 2024 to identify our population and the patients’ clinical and sociodemographic characteristics. A total of 214 patients were identified. Of these, 160 patients were excluded because their records were incomplete, they were transferred to other hospitals after stabilization, or they were deemed ineligible for reperfusion therapy due to their young age.

Finally, 64 patients were included. They were classified by the affected vascular territory, specifically posterior circulation infarction (*n* = 24; 37.5%) and anterior circulation infarction (*n* = 40; 62.5%).

### 2.1. Clinical and Sociodemographic Characteristics

The characteristics of both groups are shown in Table 1 and Table 2. The mean age of our patients was 39.4 ± 11.9 years. In our study, we defined young patients as those under 40 years of age. As shown in Table 1, the most frequent risk factors were Arterial Hypertension (34.4%) and type 2 diabetes (21.9%).

A statistically significant difference was identified in patients diagnosed with hypertension and the vertebrobasilar territory; hypertension was more common among patients with anterior circulation infarction (*p* = 0.019).

Only 53.1% of patients completed the diagnostic approach. The main cause of the stroke was cardioembolism. Atherosclerosis was identified only in patients with anterior stroke. In contrast, arterial dissection was identified only in patients with posterior circulation stroke (Table 2).

The most common symptoms in both groups were facial paralysis, dysarthria, and headache. Patients’ initial symptoms showed statistically significant differences in terms of the area involved, except for dysarthria.

The National Institutes of Health Stroke Severity Scale (NIHSS) score on admission showed a median score of 10 (3 to 16). Table 2 shows that only 63 patients underwent this evaluation; the patients were categorized as having a minor stroke (NIHSS < 5 points), moderate stroke (NIHSS 5 to 15 points), moderate to severe stroke (NIHSS 16 to 20 points), and severe stroke (NIHSS > 20 points).

Only 21.9% (n = 14) of patients with AIS received reperfusion therapy. The primary treatment was recombinant tissue-type plasminogen activator (n = 13), and 10 patients had anterior circulation infarction (*p* = 0.190).

The functional prognosis of the patients was determined using the modified Rankin Scale (mRS). Patients with an excellent functional prognosis (mRS = 0–1) were 27 (42.2%), 2 (7.8%) had a good prognosis (mRS = 2), and 22 (34.4%) had a poor functional prognosis (mRS = 3–5).

At 90 days, the percentage of patients with an excellent mRS increased to 48.5% (n = 31), and the poor functional prognosis decreased to 26.6% of patients (n = 17), as shown in Table 2. The forecast was compared according to territory, and no statistically significant differences were found.

### 2.2. Gene Expression in Patients with Infarction

Gene expression of JAK2, STAT3, and PDL1, as well as miRNA-216a and miRNA-155, was evaluated as a prospective exploratory sub-analysis in 23 patients within the first 24 h of hospital admission, before any pharmacological intervention, between May 2023 and May 2024. These patients were compared with 23 age- and sex-matched healthy individuals. All healthy participants were interviewed to exclude the presence of acute or chronic illnesses, as well as the use of medications, drugs, alcohol, or tobacco.

The mean age of the stroke population was 36.7 ± 11.2 years, while the control group was 33.5 ± 6.9 years (*p* = 0.24). The proportion of men and women did not differ between the two groups (*p* = 0.55): 14 men and 9 women were included in the stroke population, and 11 men and 12 women in the control group.

Gene expression levels were measured using the average fold change using relative quantification (ΔCt). Table 3 compared gene expressions in patients with stroke versus HP. Patients with AIS showed overexpression of all genes compared to the control group, except miRNA-216a. In contrast, no statistically significant differences were found when comparing gene expression by territory.

**Figure 1:**
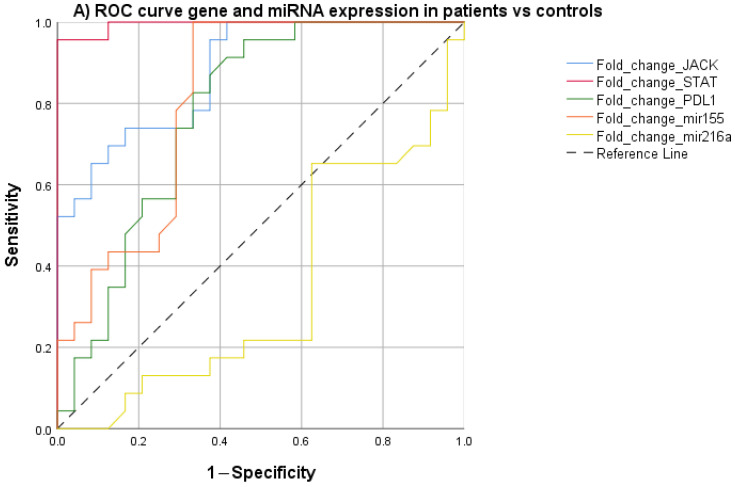

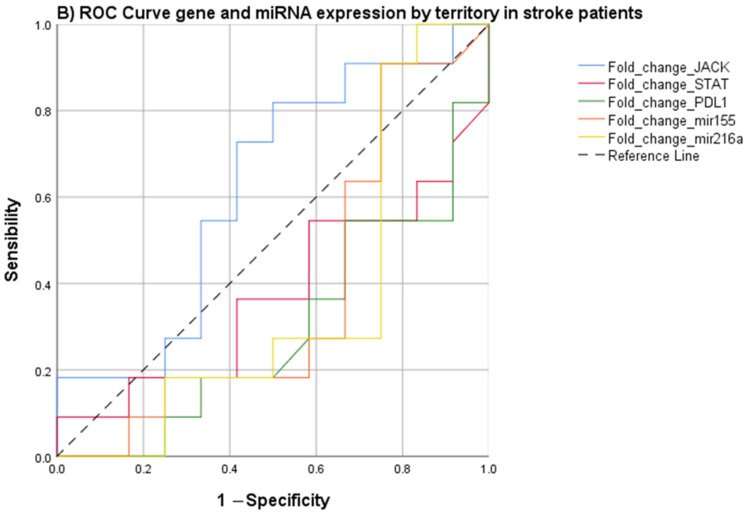
ROC curve for JAK2, STAT3, PDL-1, miR-155, miR-216a gene expression in Acute Ischemic Stroke; (**A**) patients vs. healthy population, (**B**) posterior vs. anterior circulation infarction.

The exploratory diagnostic performance of JAK2, STAT3, PD-L1, and miR-155 was assessed using receiver operating characteristic (ROC) curve analysis to evaluate their ability to discriminate patients with acute ischemic stroke from the HP. In this exploratory analysis, STAT3 showed the highest area under the curve, with a cut-off value of 3.02, corresponding to a sensitivity of 95.7% and a specificity of 100%. JAK2, PD-L1, and miR-155 demonstrated more modest discriminative performance. The JAK2 cutoff of 2.69 had a sensitivity = 73.9% and a specificity = 78.3%. The PDL-1 cutoff of 1.39 had a sensitivity = 65.2% and a specificity = 73.9%, and the miRNA 155 cutoff of 1.63 had a sensitivity = 56.5% and a specificity = 69.6%, as shown in Figure 1A and Table 4.

However, in the ROC curve analysis, the genetic markers did not demonstrate discriminative ability to identify or predict anterior or posterior cerebral infarction, without showing a statistically significant relationship between these markers and the territory of the cerebral infarction (Figure 1B and Table 4).

In addition, fold-change levels were compared across all risk factors, symptoms, complications, and locations. Only those showing statistically significant differences are shown in Table 5.

JAK2 levels were higher in smoking patients (*p* = 0.006); elevated results were also observed in the presence of symptoms such as facial paralysis (*p* = 0.007) and ataxia (*p* = 0.009). Statistically significant differences were found when comparing elevated STAT3 levels and patients undergoing tracheostomy (*p* = 0.008), and the stroke was in the posterior cerebral artery (*p* = 0.030).

PD-L1 levels were higher in patients with weakness (*p* = 0.049), and strokes in the middle cerebral artery (*p* = 0.016). However, they were lower in those with strokes in the posterior cerebral artery (*p* = 0.046).

miRNA-155 showed higher levels in patients who started with symptoms of aphasia (*p* = 0.006) and facial paralysis (*p* = 0.034). When comparing miRNA-216a levels, lower levels were observed in patients with stroke involving the anterior cerebral artery (*p* = 0.032).

## 3. Discussion

Our study presents epidemiological and clinical characteristics, as well as the outcome of patients with AIS treated in our hospital. We also sought to analyze gene expression as a potential biomarker in acute ischemic stroke.

Among the general characteristics of our study, we can observe that the average age of our population was 39.4 years. This does not correspond to what has been reported in other studies [10,11], due to the care criteria of our hospital.

Regarding the affected area, our study presents a higher proportion of patients with posterior infarction (37.5%) compared to that reported by Zeng et al. 2015 and Huo et al. 2020 (21.5% and 19.6%, respectively) [12,13].

Posterior circulation infarction occurred more frequently in young patients (<40 years). Posterior circulation infarction is most common in the youngest age groups [14].

Arterial hypertension was the only risk factor that we found to have statistically significant differences (*p* = 0.019), which is more common in anterior circulation infarction. Our results were contradictory; hypertension was described as a risk factor with no differences between the two groups. However, diabetes mellitus is a factor that confers a greater risk in posterior circulation infarction than in anterior circulation infarction [15].

The main identified cause of the stroke was cardioembolic (15.6%). Most ischemic strokes are secondary to cardioembolic, which results in a more debilitating disease and has higher rates of early recurrence in comparison [16].

Patients’ initial symptoms found in these patients are common [17]. Some symptoms favor the differential diagnosis between posterior and anterior infarction. Posterior infarction is characterized by crossed motor/sensory deficits, oculomotor nerve palsy, visual field deficits, vertigo, nausea, vomiting, and headaches [18].

Most of our patients had a minor to moderate severity of stroke, as assessed by the NIHSS. However, the severity categorization of strokes may be underestimated on the NIHSS, as the scale prioritizes symptoms related to the anterior circulation. Therefore, it has been shown that posterior circulation infarcts are more likely to be categorized as minor infarcts.

In our study, the percentage of patients who received reperfusion therapy with pharmacological thrombolysis was considerably higher than that reported in our country (21.9% vs. 7.6%) [19].

An excellent functional prognosis was observed in a greater proportion of patients with posterior circulation cerebral infarction, and a poor functional prognosis in a greater proportion of patients with anterior circulation infarction; however, this did not represent a statistically significant difference.

The prognosis of anterior circulation infarction is worse than that of posterior circulation infarction [20]. This could be an explanation for the higher number of deaths in patients with anterior compared to posterior circulation infarction (22.5% vs. 4.2%). The second evaluation of the prognosis after 90 days showed an increase in excellent prognosis and a decrease in poor prognosis.

### Analysis of Gene Expression in Stroke Patients

Furthermore, gene expression was assessed in stroke patients and compared with that of a control group, revealing overexpression of JAK2, STAT3, PDL1, and miRNA-155 in the stroke group.

The JAK2/STAT3 pathway may contribute to brain damage by affecting the expression of cytokines, such as TNF-α and IL-6, which cause inflammation [6]. JAK levels were higher in smokers; a previous study showed that smokers with ischemic stroke exhibit overexpression of the JAK-STAT pathway, which generates inflammation and can promote thrombosis and brain oxidative stress [21].

JAK-STAT is primarily expressed in various brain regions and is involved in oligodendrocyte development. JAK2 may stimulate astrocyte growth, and its alteration could be related to neurodegenerative diseases [22]. Therefore, that could explain the overexpression of JAK2 in patients who presented with facial paralysis and ataxia.

Mice with attenuated endothelial STAT3 have been observed to suffer more severe strokes. STAT3 is vital for maintaining cerebrovascular integrity, contributing to the survival and function of endothelial cells and protection against cerebral ischemia [23]. Its association with more severe strokes could explain the need for early tracheostomy.

The PDL-1 pathway is a mediator of the inflammatory response that stimulates T cells and generates the subsequent production of IL-10 [24]. Deficient PD-L1 signaling has only been shown to be associated with post-stroke immune dysregulation and poor neurological recovery. PD-L1 has a late termination of T cell activation after stroke [25]. No studies have linked PD-L1 expression to muscle weakness; however, it is regularly observed in stroke patients. This suggests a neuroinflammatory origin.

miR-155 is one of the proinflammatory miRNAs and an essential factor in macrophage activation [8]. miR-155 is a potent regulator that could modulate cell damage and limit the rapid progression of disability during stroke [26].

In contrast, our study showed no statistically significant differences when comparing miR-216a in HP and AIS patients. It has been suggested that miR-216a has a neuroprotective effect in the ischemic brain and negatively regulates the JAK2/STAT3 pathway. However, it has also been reported that miR-216a levels are low on the first day after ischemic reperfusion [27]. In our study, expression levels were measured within 24 h of stroke, so low levels of miR-216a and high levels of JAK2/STAT3 were expected. Continuous monitoring is required to observe the behavior of these genes during the different stages of the disease. This temporal dissociation between neuroprotective microRNA regulation and inflammatory signaling has been previously described in ischemic stroke [26]. In the present study, gene expression was assessed within the first 24 h after stroke onset, which may not capture this delayed upregulation. This represents an important limitation of the study and underscores the need for longitudinal analyses with serial sampling to better characterize the temporal dynamics of miR-216a during ischemic stroke.

Statistically significant differences were observed when comparing some etiologies, complications, and location of damage with some levels of gene expression; however, we also observed that the groups are heterogeneous, so more studies are needed to be conclusive.

Analysis of STAT3 expression demonstrated its potential utility as a biomarker for patients with acute stroke. In contrast, JAK2, PDL-1, and miRNA 155 had lower sensitivity and specificity. However, more in-depth analysis is needed, including a larger number of patients and comparing them with diagnosis, duration of care, prognosis, complications, systemic effects, and other inflammatory biomarkers like cytokines. In contrast, gene expression levels did not show utility in determining the affected territory in stroke.

## 4. Materials and Methods

An ambispective analytical study was conducted. A non-probabilistic sample included patients with a diagnosis of acute-phase cerebral infarction treated in the Neurology Department of the Specialty Hospital, National Medical Center of the West (IMSS), Mexico. Clinical and sociodemographic data were collected from May 2021 to May 2024. Patients treated between May 2021 and April 2023 were included retrospectively, and biological samples were unavailable for this period. Gene expression analysis was therefore conducted as a prospective sub-analysis in a subset of patients enrolled between May 2023 and May 2024, when biological sample collection was implemented.

### 4.1. Sample Collection and RNA Isolation

Peripheral blood samples (5 mL) were collected in EDTA-coated tubes from patients included in the prospective gene expression sub-analysis within the first 24 h of hospital admission and before any pharmacological intervention, between May 2023 and May 2024. Blood samples from age- and sex-matched healthy individuals were obtained during the same period.

All procedures were approved by the institutional ethics committee, and written informed consent was obtained from all participants. Total RNA, including small RNAs, was extracted from 200 µL of whole blood using the miRNeasy Mini KQiagen, Hilden, Germany), following the manufacturer’s protocol. RNA purity and concentration were assessed using a NanoDrop 2000 spectrophotometer (Thermo Fisher Scientific, Waltham, MA, USA), and RNA integrity was confirmed by agarose gel electrophoresis. Only samples with an A260/A280 ratio between 1.8 and 2.1 were included in the analysis.

### 4.2. cDNA Synthesis

For mRNA expression analysis of JAK2, STAT3, and PD-L1, reverse transcription was carried out using the High-Capacity cDNA Reverse Transcription Kit (Applied Biosystems, Foster City, CA, USA) with 500 ng of total RNA in a 20 µL reaction volume. For miRNA targets, including miR-155 and miR-216a, cDNA was synthesized from 10 ng of total RNA using the TaqMan^TM^ MicroRNA Reverse Transcription Kit (Applied Biosystems, Foster City, CA, USA) with target-specific stem-loop primers.

### 4.3. Quantitative Real-Time PCR (qRT-PCR)

Quantitative real-time PCR was performed using TaqMan^TM^ Gene Expression Assays (Hs01078136_m1 for JAK2, Hs00374280_m1 for STAT3, and Hs00204257_m1 for PD-L1) (Applied Biosystems, Foster City, CA, USA) and TaqMan^TM^ MicroRNA Assays (002623 for miR-155 and 000514 for miR-216a) (Applied Biosystems, Foster City, CA, USA). Reactions were prepared using TaqMan^TM^ Universal PCR Master Mix (Applied Biosystems, Foster City, CA, USA) and run in triplicate on a StepOnePlus^TM^ Real-Time PCR System (Applied Biosystems, Foster City, CA, USA) under the following cycling conditions: 95 °C for 10 min, followed by 40 cycles of 95 °C for 15 s and 60 °C for 1 min. The assay showed efficiency (100%) and determination coefficient (R^2^ = 0.986).

### 4.4. Normalization and Relative Quantification

GAPDH (Assay ID: Hs02758991_g1) was used as the endogenous control for mRNA normalization, and miR-16 (Assay ID: 000391) was used as the reference gene for miRNA normalization, based on its reported stability in peripheral blood. Relative quantification of gene expression was calculated using the comparative Ct (2^−ΔΔCt^) method. ΔCt was defined as the difference between the Ct value of the target gene and that of the endogenous control, and ΔΔCt was calculated by comparing ΔCt values of stroke patients to those of the control group.

### 4.5. Quality Control and Assay Validation

All reactions were conducted in technical triplicate. Negative controls without template and no-reverse transcription controls were included in each run to monitor contamination and ensure amplification specificity. PCR efficiency was assessed using a five-point serial dilution curve; assays were considered acceptable if they showed an amplification efficiency between 90% and 110% and an R^2^ ≥ 0.98. Due to the use of hydrolysis probes, no melting curve analysis was required.

### 4.6. Statistical Analysis

Categorical variables were expressed as frequencies and percentages and compared between anterior and posterior circulation infarction groups using the Chi-square test or Fisher’s exact test, as appropriate. Continuous variables were summarized as means ± standard deviation or medians with interquartile ranges and compared using Student’s *t*-test or the Mann–Whitney U test, depending on data distribution. For gene and miRNA expression analysis, ΔCt values were compared between stroke patients and healthy controls using the Mann–Whitney U test. The diagnostic performance of each biomarker was assessed using the Receiver Operating Characteristic (ROC) curve analysis. For each gene or miRNA, the area under the curve (AUC), sensitivity, specificity, and optimal cut-off value were calculated. A *p*-value < 0.05 was considered statistically significant. Statistical analyses were performed using SPSS version 28 (IBM Corp., Armonk, NY, USA).

## 5. Conclusions

Patients with AIS showed overexpression of all genes compared to the healthy population, except for miRNA-216a. The JAK2, STAT3, PDL-1, and miR-155 genes may be useful biomarkers for patients with AIS, but longitudinal analysis with a larger sample is required.

## Figures and Tables

**Table 1:** Comparison of sociodemographic characteristics of young adults with anterior circulation vs. posterior circulation infarction in patients with Acute Ischemic Stroke.

Characteristics	Total N = 64	Posterior Circulation Infarction *n* = 24	Anterior Circulation Infarction *n* = 40	*p* Value *
Sex n (%)				
Female	26 (40.6%)	7 (29.2%)	19 (47.5%)	0.118
Male	38 (59.4%)	17 (70.8%)	21 (52.5%)	
Age				
≤40 years	30 (46.9%)	15 (62.5%)	15 (37.5%)	0.046 *
>40 years	34 (53.1%)	9 (37.5%)	25 (62.5%)	
Risk Factors				
Hypertension	22 (34.4%)	4 (16.7%)	18 (45.0%)	0.019 *
Diabetes mellitus type II	14 (21.9%)	3 (12.5%)	11 (27.5%)	0.137
Dyslipidemia	3 (4.7%)	1 (4.2%)	2 (5.0%)	0.686
Atrial fibrillation	2 (3.1%)	1 (4.2%)	1 (2.5%)	0.613
Smoke	19 (29.7%)	8 (33.3%)	11 (27.5%)	0.413
Stroke/Ischemic Attack, Transient	7 (10.9%)	1 (4.2%)	6 (15.0%)	0.178
Illicit Drugs	8 (12.5%)	4 (16.7%)	4 (10.0%)	0.341

* *p*-value was calculated comparing the Posterior circulation infarction group with the Anterior circulation infarction group. * *p* < 0.05.

**Table 2:** Comparison of clinical characteristics of Young Adults with Anterior circulation vs. Posterior circulation infarction in patients with Acute Ischemic Stroke.

Characteristics	Total N = 64	Posterior Circulation Infarction *n* = 24	Anterior Circulation Infarction *n* = 40	*p* Value
Etiology of AIS				
Cardioembolism	10 (15.6%)	2 (8.3%)	8 (20.0%)	0.017 *
Atherosclerosis	5 (7.8%)	0 (0%)	5 (12.5%)	
Small vessel disease	5 (7.8%)	1 (4.2%)	4 (10.0%)	
Arterial dissection	5 (7.8%)	5 (20.8%)	0 (0%)	
Cryptogenic	9 (14.1%)	4 (16.7%)	5 (12.5%)	
Other/without diagnosis	30 (46.9%)	12 (50.0%)	18 (45.0%)	
Initial Symptoms Diagnostic				
Vertigo	13 (20.3%)	12 (50.0%)	1 (2.5%)	<0.0001 *
Nausea/vomiting	16 (25.0%)	12 (50.0%)	4 (10.0%)	0.001 *
Headache	20 (31.3%)	13 (54.2%)	7 (17.5%)	0.003
Visual disturbances	7 (10.9%)	6 (25.0%)	1 (2.5%)	0.009
Dysarthria	22 (34.4%)	7 (29.2%)	15 (37.5%)	0.344
Facial paralysis	32 (50.0%)	5 (20.8%)	27 (67.5%)	<0.0001 *
Weakness	41 (64.1%)	7 (29.2%)	34 (85.0%)	<0.0001 *
Aphasia	17 (26.6%)	7 (8.3%)	15 (37.5%)	0.009
Initial evaluation NIHSS	63 (100%)	23 (36.5%)	40 (63.5%)	
Minor stroke	21 (33.33%)	14 (60.9%)	7 (17.5%)	0.005 *
Moderate stroke	26 (41.3%)	6 (26.1%)	20 (50.0%)	
Moderate-severe stroke	6 (9.5%)	1 (4.3%)	5 (12.5%)	
Severe	10 (15.9%)	2 (8.7%)	8 (20.0%)	
Complications after AIS				
Cerebral edema	14 (21.9%)	2 (8.3%)	12 (30.0%)	0.039 *
Pneumonia	10 (15.6%)	3 (12.5%)	7 (17.5%)	0.438
Mechanical ventilation	14 (21.9%)	4 (16.7%)	10 (25.0%)	0.347
Death	10 (15.6%)	1 (4.2%)	9 (22.5%)	0.049 *
Prognosis				
Excellent functional prognosis	31	16	15	0.125
Good prognosis	2	1	1	
Poor functional prognosis	17	6	11	
Dead	10	1	9	

*p*-value was calculated comparing the Posterior circulation infarction group with the Anterior circulation infarction group. * *p* < 0.05.

**Table 3:** Comparison of the expression of genes and miRNAs in Young Adults with Acute Ischemic Stroke.

Gene Expression	Comparison of HP vs. Patients			Comparison by Territory		
	HP (*n* = 23) Mean ± SD	Stroke Patient (*n* = 23) Mean ± SD	*p*	Posterior Circulation Infarction (*n* = 11) Mean ± SD	Anterior Circulation Infarction (*n* = 12) Mean ± SD	*p*
Fold JAK2	1.71 ± 0.22	3.64 ± 0.21	<0.001 *	3.90 ± 1.00	3.42 ± 1.08	0.347
Fold STAT3	1.78 ± 0.13	3.82 ± 0.13	<0.001 *	3.72 ± 0.67	3.93 ± 0.60	0.316
Fold PDL-1	0.80 ± 0.18	1.67 ± 0.13	<0.001 *	1.43 ± 0.64	1.89 ± 0.62	0.091
Fold miRNA-155	1.18 ± 0.13	2.06 ± 0.15	<0.001 *	1.85 ± 0.53	2.26 ± 0.91	0.316
Fold miRNA-216a	1.57 ± 0.05	1.41 ± 0.05	0.061	1.35 ± 0.24	1.48 ± 0.32	0.260

HP: Healthy population. * *p* < 0.05.

**Table 4:** ROC analysis of gene expression scores in comparison of the healthy population vs. AIS patients and comparison by territory.

Gene Expression		95.0% Confidence Interval		*p* Value	Sensitivity (%)	Specificity (%)
	AUC	Lower Bound	Upper Bound			
Healthy Population vs. AIS Patients						
Fold JAK2	0.880	0.765	0.967	<0.001 *	73.9	78.3
Fold STAT3	0.995	0.986	1.000	<0.001 *	95.7	100
Fold PDL-1	0.777	0.648	0.924	<0.001 *	65.2	73.9
Fold miRNA-155	0.812	0.672	0.935	<0.001 *	56.5	69.6
Fold miRNA-216a	0.342	0.159	0.472	0.064	-	-
*Posterior* vs. *Anterior Circulation Infarction*						
Fold JAK2	0.621	0.385	0.858	0.325	-	-
Fold STAT3	0.375	0.138	0.612	0.310	-	-
Fold PDL-1	0.292	0.078	0.506	0.091	-	-
Fold miRNA-155	0.375	0.134	0.616	0.310	-	-
Fold miRNA-216a	0.356	0.113	0.599	0.242	-	-

AUC: area under the curve; * *p* < 0.05.

**Table 5:** Fold change in gene expression compared with risk factors in stroke patients.

Characteristics Fold Mean ± SD	JAK2	STAT3	PDL-1	miRNA-155	miRNA-216a
Etiology					
Smoke					
Yes (n = 11)	4.21 ± 0.97	3.75 ± 0.56	1.78 ± 0.62	1.88 ± 0.69	1.44 ± 0.26
Not (n = 12)	3.13 ± 0.84	3.89 ± 0.71	1.56 ± 0.70	2.24 ± 0.82	1.39 ± 0.30
(*p*-value)	(0.006 *)	(0.608)	(0.379)	(0.260)	(0.695)
Aphasia					
Yes (n = 7)	3.46 ± 1.13	3.95 ± 0.64	1.77 ± 0.67	2.64 ± 1.01	1.34 ± 0.39
Not (n = 16)	3.73 ± 1.03	3.77 ± 0.64	1.62 ± 0.97	1.81 ± 0.46	1.45 ± 0.23
(*p*-value)	(0.671)	(0.624)	(0.624)	(0.006 *)	(0.624)
Weaknesses					
Yes (n = 13)	3.59 ± 0.98	3.92 ± 0.58	1.90 ± 0.54	2.19 ± 0.90	1.47 ± 0.30
Not (n = 10)	3.72 ± 1.17	3.71 ± 0.71	1.36 ± 0.63	1.90 ± 0.54	1.34 ± 0.25
(*p*-value)	(0.927)	(0.313)	(0.049 *)	(0.738)	(0.313)
Facial paralysis					
Yes (n = 15)	3.18 ± 0.96	3.79 ± 0.59	1.82 ± 0.61	2.30 ± 0.86	1.41 ± 0.31
Not (n = 8)	4.52 ± 0.50	3.90 ± 0.73	1.38 ± 0.68	1.62 ± 1.58	1.42 ± 0.23
(*p*-value)	(0.007 *)	(0.728)	(0.169)	(0.034 *)	(0.776)
Ataxia					
Yes (n = 4)	4.81 ± 0.43	4.03 ± 0.45	1.21 ± 0.51	1.70 ± 0.20	1.52 ± 0.11
Not (n = 19)	3.40 ± 0.97	3.78 ± 0.66	1.77 ± 0.65	2.14 ± 0.82	1.39 ± 0.30
(*p*-value)	(0.009 *)	(0.456)	(0.138)	(0.667)	(0.907)
Complication					
Tracheostomy					
Yes (n = 2)	3.38 ± 1.43	4.88 ± 0.35	2.00 ± 0.14	2.16 ± 0.93	1.35 ± 0.42
Not (n = 21)	3.67 ± 1.04	3.73 ± 0.56	1.64 ± 0.68	2.06 ± 0.77	1.42 ± 0.28
(*p*-value)	(0.711)	(0.008 *)	(0.443)	(0.791)	(0.957)
Location					
MCA					
Yes (n = 6)	2.92 ± 0.91	3.78 ± 0.62	2.20 ± 0.38	1.74 ± 0.26	1.59 ± 0.10
Not (n = 17)	3.90 ± 0.98	3.84 ± 0.65	1.48 ± 0.63	2.18 ± 0.85	1.35 ± 0.30
(*p*-value)	(0.044)	(0.973)	(0.016 *)	(0.392)	(0.074)
ACA					
Yes (n = 2)	3.65 ± 1.81	4.73 ± 0.18	2.35 ± 0.35	2.50 ± 0.45	0.99 ± 0.78
Not (n = 21)	3.65 ± 1.02	3.74 ± 0.59	1.61 ± 0.65	2.02 ± 0.78	1.46 ± 0.26
(*p*-value)	(0.870)	(0.071)	(0.126)	(0.158)	(0.032 *)
PCA					
Yes (n = 5)	3.85 ± 0.93	3.29 ± 0.39	1.12 ± 0.63	1.81 ± 0.46	1.24 ± 0.20
Not (n = 18)	3.59 ± 1.09	3.98 ± 0.61	1.82 ± 0.59	2.13 ± 0.83	1.46 ± 0.29
(*p*-value)	(0.914)	(0.030 *)	(0.046 *)	(0.801)	(0.055)

MCA, Middle cerebral artery; ACA, Anterior cerebral artery; PCA, Posterior cerebral artery; * *p* < 0.05.

## Data Availability

The data presented in this study are available on request from the corresponding author (provided that this does not compromise patient privacy or data integrity).

## Abbreviations

The following abbreviations are used in this manuscript:

ΔCt	change using relative quantification
AIS	Acute ischemic stroke
AUC	area under the curve
HP	Healthy Population
mRS	modified Rankin Scale (mRS)
NIHSS	National Institutes of Health Stroke Severity Scale
ROC	Receiver Operating Characteristic
